# Lacritin Salvages Human Corneal Epithelial Cells from Lipopolysaccharide Induced Cell Death

**DOI:** 10.1038/srep18362

**Published:** 2015-12-16

**Authors:** Venkat Rao Vantaku, Geetika Gupta, Krishna Chaitanya Rapalli, Roy Karnati

**Affiliations:** 1 School of Life Sciences, University of Hyderabad, Gachibowli, Hyderabad, India 500046

## Abstract

Innate immunity of the corneal epithelium is conferred by proteinaceous secretions from the epithelium and associated lacrimal and meibomian glands. Lacritin, an eye-specific protein with anti-microbial, cytoprotective and wound-healing properties, predominantly secreted by lacrimal glands, is absent in conditions such as Dry eye and Keratitis. In view of the biological significance of lacritin in human eye, we investigated its role in human corneal epithelial (HCE) cells during lipopolysaccharide (LPS)-induced infection. LPS-challenged HCE cells demonstrated apoptosis-mediated cell death and elevated lacritin levels. The LPS-induced cell death is alleviated with exogenous supplementation of recombinant lacritin. This cytoprotective effect of lacritin is mediated through Cyclooxygenase-2 (COX-2). This study is the first to highlight the protective role of lacritin and mechanism of its action during bacterial infection of cornea *in vitro*.

Eye infection is one of the major causes of visual impairment and blindness^1^. Well-conserved structural motifs of different microorganisms including LPS of the gram-negative bacteria can mediate innate immune responses leading to either activation or suppression of inflammatory processes and eventually cell death^2^. LPS administration increased the number of apoptotic cells in corneal injury models^3^ and induced the expression of autophagic related genes^4^. LPS, through its receptor, toll-like receptor 4 (TLR4), can induce cell migration, proliferation and wound healing^5^.

Innate immune response to ocular infection involves several factors such as lysozyme, lipocalin, lactoferrin, mucins, surfactant protein D, secretory IgA, cytokeratin-derived antimicrobial peptides, β-defensins, constitutively-expressed tear proteins and numerous uncharacterized secretory proteins from lacrimal and meibomian glands and from the corneal and conjunctival epithelium that are activated upon infection^6^,^7^,^8^,^9^,^10^,^11^. Elucidating the role of these proteins in ocular innate defenses is critical in designing effective therapeutic strategies. Expression of several proteins, including extracellular ones^11^, are altered with eye pathologies^12^,^13^,^14^,^15^,^16^. Human tear glycoprotein, lacritin, is the only molecule with mitogenic potential^17^ and anti-microbial activity^18^,^19^, which is down regulated in Dry eye^14^, Keratitis^20^, and various other pathological conditions associated with ocular tissue^12^,^16^. In addition, lacritin induces tear secretion^21^,^22^, relieves epithelial stress^23^, offers cytoprotection^24^, and promotes corneal wound-healing^25^. Thus, this multifunctional eye-specific factor with its potential role(s) in corneal integrity has immense therapeutic value.

In the present study, we demonstrate that lacritin plays a ‘saviour’ role during LPS-induced corneal infection. Furthermore, we obtained the cues to decipher the underlying mechanism by which lacritin might confer innate immunity to the cells.

## Results

### LPS induces apoptosis-mediated corneal cell death

**Figure 1:**
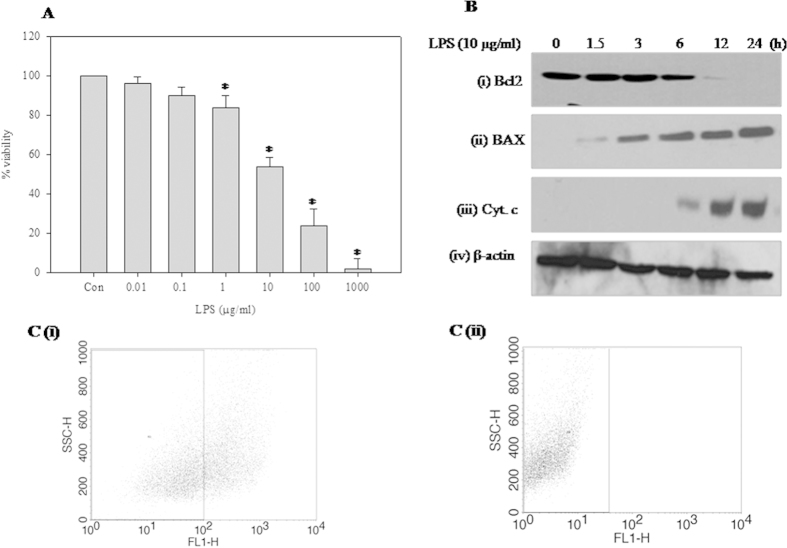
Effect of LPS on HCE cells. (**A**) MTT cell viability assay. HCE cells were treated with various concentrations of LPS (0.01, 0.1, 1, 10, 100, and 1000 μg/ml) for 24 h. Viability of untreated control cells was 100% and it decreased with increase in LPS concentration administered for 24 h. Data is expressed as mean percent of untreated control ± SEM for three independent experiments. *Indicates significant difference at p < 0.05 when compared to untreated control. (**B**) Effect of LPS on (**i**) Bcl-2 (**ii**) Bax and (**iii**) Cyt c release (**iv**) β-actin was used as an internal control. HCE cells were treated with LPS (10 μg/ml) for different time periods (0, 1.5, 3, 6, 12 and 24 h). Equal amounts of protein was analyzed by SDS–PAGE (10–12%), proteins on the gel were transferred on to nitrocellulose membrane and probed with protein-specific antibody under same experimental conditions. The representative images of three independent experiments shown were cropped. (**C**) Effect of LPS on mitochondrial membrane potential. HCE cells were treated with LPS (10 μg/ml) for 24 h and incubated with Rhodamine 123 for 20 min and fluorescence was quantified by FACS on FLH-1. (**i**) Control and (**ii**) LPS (10 μg/ml) treated.

LPS treatment caused death of human corneal epithelial cells in a dose-dependent manner. Cell death induced by lower doses of LPS (<1 μg/ml) was not significantly different from the control; however, 50% cell death was observed at a dose of 10 μg/ml LPS. Further high dosage (>10 μg/ml) resulted in extensive cell death (Fig. 1A). To further validate the cell death, Bcl2 and Bax expression was monitored. Expression of Bcl2 was inhibited (Fig. 1B i), and Bax (Fig. 1B ii) activated, in a time-dependent manner when the cells were treated with an IC_50_ dose of LPS. Increased release of Cytochrome c (Cyt c) from the mitochondrial membrane spaces into the cytoplasm upon LPS treatment further confirms apoptosis-mediated cell death (Fig. 1B iii). Change in mitochondrial membrane potential, which is also a measure of apoptotic cell death, was studied by flow cytometry analysis using Rhodamine 123. LPS-induced mitochondrial membrane depolarization was depicted by the shift in cell population towards the lower scale of the FL1-H. In comparison to control cells (Fig. 1C i), 42% LPS-treated cells were depolarized (Fig. 1C ii).

### Corneal epithelial cell signaling is altered by LPS

**Figure 2:**
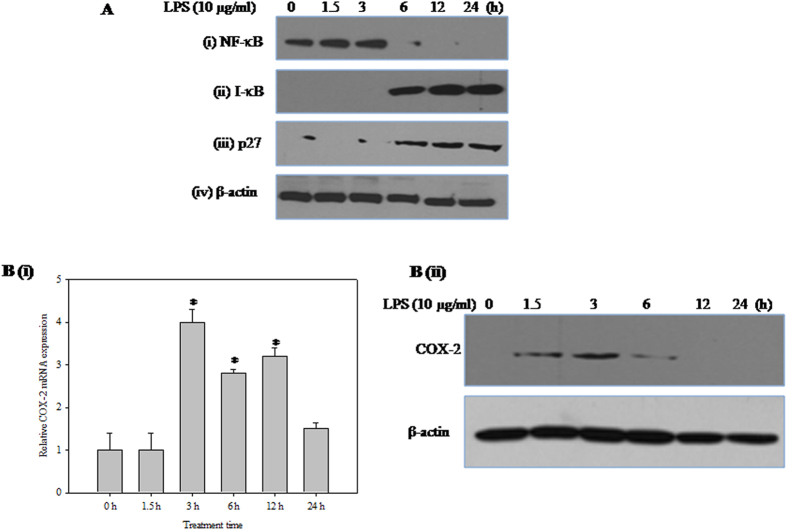
Effect of LPS on corneal epithelial cell signaling. (**A**) Effect of LPS on the levels of (**i**) NF-κB-p65 (**ii**) I-κBα and (**iii**) p27 proteins (**iv**) β-actin was used as an internal control. HCE cells were treated with LPS (10 μg/ml) for different time periods (0, 1.5, 3, 6, 12 and 24 h). Equal amounts of total protein (for I-κB α, p27 and β-actin) and nuclear protein (for NF-κB-p65) was analysed by SDS-PAGE (10–12%), and after electrophoresis under same experimental conditions, proteins on the gel were transferred on to nitrocellulose membrane and probed with specific antibodies. The representative images of three independent experiments shown were cropped. (**B**) Effect of LPS on the expression of COX-2. (**i**) Quantitative analysis of COX-2 mRNA expression in HCE cells relative to 18S rRNA expression at different time points (0, 1.5, 3, 6, 12 and 24 h) after LPS treatment by qRT-PCR is reported as fold change relative to 0 h calculated using 2^−ΔΔCT^. X-axis represents exposure time in hours. Values are mean ± SEM for three independent experiments. *Indicates significance at p < 0.05. (**ii**) HCE cells were treated with LPS (10 μg/ml) for different time periods (0, 1.5, 3, 6, 12 and 24 h). Equal amounts of protein was analyzed by SDS–PAGE (12%), proteins on the gel were transferred on to nitrocellulose membrane and probed with COX-2 specific antibody under same experimental conditions. The representative images of three independent experiments shown were cropped.

The cellular dynamics between nuclear factor kappa-light-chain-enhancer of activated B cells (NF-κB) and nuclear factor of kappa light polypeptide gene enhancer in B-cells inhibitor-alpha (I-κB α) is altered in LPS-treated corneal epithelial cells. LPS-treated nuclear lysates showed time-dependent decrease in the nuclear translocation of NF-κB (Fig. 2A i). In addition, elevated I-kB α levels indicate inhibition of its degradation in the whole cell extracts (Fig. 2A ii). Furthermore, the expression of the cyclin-dependent kinase inhibitor, p27, a key regulator of cell proliferation and apoptosis was also increased with LPS treatment (Fig. 2A iii). Furthermore, the effect of LPS treatment on COX-2 expression at both mRNA (Fig. 2B i) and protein level was (Fig. 2B ii) monitored using real-time RT-PCR and western blot analysis respectively. The COX-2 expression was elevated at 3 h and diminished at later time points up to 24 h.

### Lacritin recovers corneal epithelial cells from the deleterious effects of LPS

**Figure 3:**
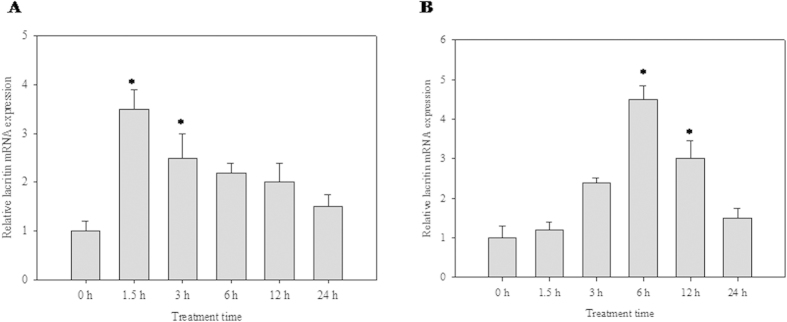
Effect of LPS on lacritin gene expression. Quantitative analysis of lacritin mRNA expression in HCE cells relative to 18S rRNA expression at different time points (0, 1.5, 3, 6, 12 and 24 h) after LPS treatment by qRT-PCR is reported as fold change relative to 0 h calculated using 2^−ΔΔCT^. X-axis represents exposure time in hours. Values are mean ± SEM for three independent experiments. *Indicates significance at P < 0.05. (**A**) LPS (10 μg/ml) (**B**) LPS (100 ng/ml).

**Figure 4:**
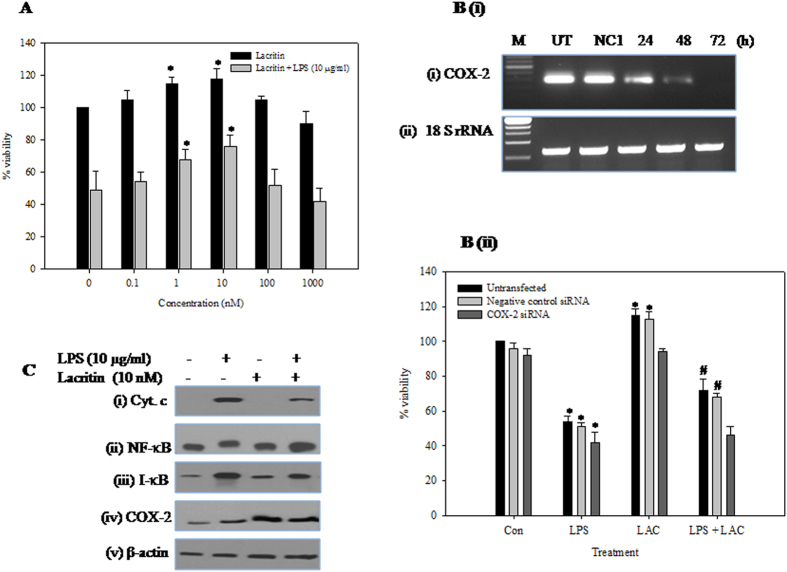
Effect of recombinant lacritin on HCE cells. (**A**) Effect of recombinant lacritin on HCE cells with/without LPS. HCE cells were treated with either lacritin alone (0. 0.1, 1, 10, 100, 1000 nM) or lacritin (0, 0.1, 1, 10, 100, 1000 nM) and LPS (10 μg/ml) for 24 h. Viability of untreated control cells was 100% and data is expressed as mean percent of untreated control ± SEM for three independent experiments. *Indicates significant difference at p < 0.05 when compared to untreated control. (**B**) Effect of COX-2 knockdown on lacritin’s cytoprotection. (**i**) Semi quantitative RT-PCR analysis of COX-2 expression in the HCE cells transfected or untransfected (UT) with NC1 or COX-2 siRNA for 24, 48 and 72 hours. 18S rRNA served as internal control. (**ii**) Untransfected or NC1 transfected or COX-2 depleted HCE cells were treated with either LPS (10 μg/ml) alone or lacritin alone (10 nM) or both for 24 h. Viability of untransfected control cells was 100% and data is expressed as mean percent of untreated control ± SEM for three independent experiments. *Indicates significant difference at p < 0.05 when compared to untreated control. #Indicates significant difference at p < 0.05 when compared to their respective LPS alone treated cells. (**C**) Effect of recombinant lacritin on the levels of (**i**) Cyt c (**ii**) NF-κB-p65 (**iii**) I-κB α and (**iv**) COX-2 in LPS treated HCE cells. HCE cells were treated with either LPS (10 μg/ml) alone or lacritin (10 nM) alone or both for 24 h. Equal amounts of total protein (for I-κBα and β-actin), nuclear protein (for NF-κB-p65) and cytosolic protein (for Cyt c) was analysed by SDS- PAGE (10–12%), and after electrophoresis under same experimental conditions, proteins on the gel were transferred on to nitrocellulose membrane and probed with protein specific antibodies. The representative images of three independent experiments shown were cropped.

LPS-treated corneal epithelial cells showed temporal regulation of lacritin. High dose of LPS (10 μg/ml) induced early expression of lacritin (i.e. at 1.5 h) (Fig. 3A), whereas a lower dose (100 ng/ml) induced late expression (Fig. 3B). Lacritin treatment (0, 0.1, 1, 10, 100, 1000 nM) increases the HCE cell number in biphasic manner with the most effective dose at 10 nM. As lacritin shows maximum effect at 10 nM and viability of HCE cells decreases to 50% at (10 μg/ml) of LPS, all the experiments were performed at these doses. Addition of recombinant lacritin (10 nM) to the LPS-treated cells increased the cell viability from 54% to 72%. Lacritin alone increased total cell number by ~15% (Fig. 4A). However, lacritin was unable to restore the cell viability of LPS treated HCE cells depleted of COX-2 (Fig. 4B). Cyt c release during LPS-mediated apoptosis was reduced (Fig. 4C i). Further, NF-κB translocation to the nucleus (Fig. 4C ii) and I-kB α degradation (Fig. 4C iii) was enhanced. Furthermore, the declined levels of COX-2 were elevated in the presence of lacritin (Fig. 4C iv).

## Discussion

Corneal epithelium in synergy with tear fluid and its soluble factors plays a crucial role in innate immunity during infection^26^. LPS, the bacterial virulence factor, is recognized by TLR4 on the epithelial cells; thereafter via downstream signaling regulate the proteins responsible for innate immunity^27^. Functional aspects of most tear proteins are uncharacterized in the eye pathophysiology^11^. Lacritin is one such eye-specific bactericidal protein^11^,^18^,^19^ whose role in corneal infection remains largely unexplored. For the first time, we demonstrated that lacritin salvages the corneal epithelium against bacterial LPS and that this effect is mediated through COX-2.

Prominent corneal epithelial cell death observed at high LPS doses in our study corroborates most of the previous studies depicting apoptosis of cornea during infection by LPS^3^,^28^. Together, these studies indicate that the LPS action on the human corneal epithelium is dose-dependent.

LPS-mediated apoptosis involves the alteration of Bcl2 family proteins^29^. The decrease in the Bcl2/Bax protein ratio is a pro-apoptotic signal which shifts the homeostasis of the cells towards death by depolarizing and permeabilizing mitochondria resulting in release of Cyt c into the cytoplasm which in turn activates further steps for the execution of cell death. Similar to our study, Bcl2/Bax expression pattern was also observed in HCE cells treated with allethrin^30^ and during hyper-osmolarity^31^.

NF-κB activation observed in the present study as well as in *Pseudomonas aeruginosa* infected corneal epithelial cells^32^ reiterates the role of NF-κB as a first-line of defense during inflammation and infection^32^,^33^. NF-κB is rapidly activated by a large spectrum of chemically diverse agents including bacterial LPS, microbial and viral pathogens. Inactive NF-κB sequestrated in the cytoplasm, is bound by I-κB family proteins. Diverse stimuli phosphorylate I-κB leading to its ubiquitination and subsequent degradation thus exposing nuclear localization signals (NLS) on NF-κB subunits. This results in translocation of NF-κB and subsequent activation of gene transcription^34^.

CDK inhibitor, P27, is a protein which plays an important role in progression of cell cycle in corneal epithelial cells^35^. Elevated levels of p27 were detected in LPS-induced corneal cells similar to that shown in staurosporine-induced apoptosis of corneal epithelial cells^36^.

In the present study, the ability of the cell to temporally regulate lacritin expression based on the LPS concentration indicates that LPS evokes innate immunity in addition to causing damage to the corneal epithelial cells as demonstrated elsewhere^37^. Whether LPS-induced lacritin confers innate immunity remains to be investigated further; however, lacritin was shown to offer cytoprotection to the human corneal epithelial cells under different stress conditions^23^,^24^.

LPS modulates COX-2 expression in a similar pattern as that of lacritin. Further, it is reported that lacritin’s potential to induce proliferation is mediated by COX-2^17^. This indicates that the enhanced expression of COX-2 at 3 h could be a result of lacritin induction at an earlier time point (i.e., at 1.5 h) in corneal cells. However, normal levels of COX-2 were restored when LPS was administered in the presence of lacritin. This restoration offers partial but significant cytoprotection. Lacritin was unable to play a cytoprotective role in the LPS treated HCE cells depleted of COX-2, suggesting that the cytoprotective role of lacritin is mediated through COX-2.

The cytoprotective role of lacritin could be mediated by COX-2, in addition to lacritin’s ability to reverse LPS-induced apoptotic signaling as shown in the present study and based on previous reports^17^,^38^. Few studies have shown the role of autophagy in cytoprotective function of lacritin^23^,^24^. Additional studies in this direction are warranted to elucidate the true therapeutic potential of this molecule in eye infections.

## Methods

### Materials

Human corneal epithelial cells were obtained from L.V. Prasad Eye Institute, Hyderabad India. T-25, T-75 flasks and culture dishes were purchased from Corning Life Sciences, USA. Fetal bovine serum (FBS) and Rhodamine 123 were procured from GIBCO-BRL Life Technologies, USA. Antibodies to Cyt c, Bcl-2, Bax, NF-κB, I-κB, p27 were purchased from Upstate Biotechnology, USA. COX-2 antibody was obtained from Cayman Chemicals, USA. COX-2 siRNA was from Santacruz Biotechnology, USA. Universal negative control (NC1) siRNA was from IDT, USA. Western blotting detection reagent was purchased from GE Healthcare Life Sciences, USA. cDNA synthesis kit and Lipofectamine 2000 was ordered from Invitrogen, USA. MEM alpha, EGF, Insulin, nutrient mixture F12, *Pseudomonas aeruginosa* LPS, MTT [3-(4,5-dimethylthiazol-2-yl)-2,5-diphenyl-2*H*-tetrazolium bromide], BCIP/NBT, TMB/H_2_O_2_, TRIZOL reagent, monoclonal antibody against β-actin were purchased from Sigma, USA. All other chemicals and reagents of molecular biology grade were purchased from local companies in India.

### Cell viability assay

Cell viability was determined by MTT assay. HCE cells were seeded in a 96-well culture plate at density of 6 × 10^3^ cells/well overnight and grown in the presence or absence of LPS (0.01, 0.1, 1, 10, 100, and 1000 μg/ml) for 24 h in a final volume of 100 μl medium. The medium was aspirated and 20 μl of MTT (5 mg/ml) was added to the fresh medium. After 3 h incubation at 37 °C, the formazan crystals were dissolved in 100 μl of DMSO. Absorbance was read at 570 nm on a multi-well plate reader. The percentage inhibition of cell viability was calculated as a fraction of control. To elucidate the cytoprotective function of lacritin and role of COX-2, lacritin (10 nM) was pre-incubated for 2 h before the addition of LPS (10 μg/ml) with HCE cells or HCE cells depleted of COX-2. For knockdown of COX-2, HCE cells were transfected with COX-2 siRNA at final concentration of 10 nM with Lipofectamine 2000 at a ratio of 1:2 for 24, 48 and 72 h. Untransfected and Universal negative control 1 siRNA transfected cells served as controls.

### Western blotting analysis

Nuclear, cytoplasmic and whole extracts were prepared from control and treated cells as described previously^39^. The protein content was estimated by the Bradford assay^40^. Proteins were resolved on 7–12% SDS-PAGE gels and transferred onto nitrocellulose or PVDF membranes. The membranes were blocked with 3% (w/v) BSA and incubated with the primary antibodies [Bax, Bcl-2, Cyt C, p27, NFκB-p65, I-κB α (1:1000 dilution) and β-actin (1:500 dilution)] in 10 ml of antibody-dilution buffer (1× Tris-buffered saline and 0.05% Tween 20 with 1% BSA) with gentle shaking at 4 °C for 8–12 h and incubated with horseradish peroxidase or alkaline phosphatase conjugated secondary antibodies. The signals were detected by using specific substrates.

### Mitochondrial membrane potential estimation by flow cytometry analysis

After treatment with LPS (10 μg/ml), cells were incubated with Rhodamine 123 (10 μg/ml) for 20 min. Data were collected using the program Cell Quest of FACS Calibur (Becton Dickinson, CA, USA) and fluorescence was measured using a FL-1 detector.

### Real-time PCR studies

Total RNA was isolated from cells using supplier’s protocol. Superscript^TM^ III first strand synthesis system was used to synthesize cDNAs. Primers were designed based on the gene sequences available in NLM nucleotide database. For each gene, sequences of the forward and reverse primers used in respective PCR, are as follows: Lacritin Fwd: 5′CTCTGACTCGACGGGTGCTG3′ Rev: 5′CCGAAGTCTCCTGGGCTGTT3′; COX-2 Fwd: 5′AACAGGAGCATCCTGAATGG3′, Rev: 5′GGTCAATGGAAGCCTG TGATG3′; 18S Fwd: 5′GCTACCACATCCAAGGAAGGCAGC3′, Rev: 5′CGGCTGCTGG CACCAGACTTG3′. Real-time studies were performed on an ABI Prism H7500 fast thermal cycler (Applied Biosystems, CA, USA). Each sample was run in triplicate in a final volume of 25 μl containing 1 μl of template (1:5 dilution), 10 pmol of each primer and 12 μl of Power SYBR Green PCR master mix (Applied Biosystems). The real-time PCR results were presented as a change in expression relative to control using target gene C_t_ values normalized to that of 18S gene C_t_ values based on the comparative C_t_ method^41^.

### Statistical analyses

The data for all the studies were obtained from three independent experiments and average was calculated for all the variables. Differences between groups were analyzed by ANOVA followed by Student Newman–Keuls test using Sigma Stat software. Values were considered significant at p < 0.05.

## Additional Information

**How to cite this article**: Vantaku, V. R. *et al.* Lacritin Salvages Human Corneal Epithelial Cells from Lipopolysaccharide Induced Cell Death. *Sci. Rep.* **5**, 18362; doi: 10.1038/srep18362 (2015).
